# Changing landscape of liver transplant in the United States—*time for a new innovative way to define and utilize the “non-standard liver allograft”—a proposal*

**DOI:** 10.3389/frtra.2024.1449407

**Published:** 2024-08-08

**Authors:** Rashmi Seth, Kenneth A. Andreoni

**Affiliations:** ^1^ Department of Surgery, Division of Transplant Surgery, University of Tennessee Health Sciences Center, Methodist University Hospital Transplant Institute, Memphis, TN, United States; ^2^ Department of Surgery, Division of Abdominal Transplantation, Thomas Jefferson University, Philadelphia, PA, United States

**Keywords:** liver allograft composite score (LACS), liver allograft variables (LAV), liver transplant, marginal donor, extended criteria donor (ECD), non-standard liver allograft, model for end stage liver disease, donation after cardiac death (DCD)

## Abstract

Since the first liver transplant was performed over six decades ago, the landscape of liver transplantation in the US has seen dramatic evolution. Numerous advancements in perioperative and operative techniques have resulted in major improvements in graft and patient survival rates. Despite the increase in transplants performed over the years, the waitlist mortality rate continues to remain high. The obesity epidemic and the resultant metabolic sequelae continue to result in more marginal donors and challenging recipients. In this review, we aim to highlight the changing characteristics of liver transplant recipients and liver allograft donors. We focus on issues relevant in successfully transplanting a high model for end stage liver disease recipient. We provide insights into the current use of terms and definitions utilized to discuss marginal allografts, discuss the need to look into more consistent ways to describe these organs and propose two new concepts we coin as “Liver Allograft Variables” (LAV) and “Liver Allograft Composite Score” (LACS) for this. We discuss the development of spectrum of risk indexes as a dynamic tool to characterize an allograft in real time. We believe that this concept has the potential to optimize the way we allocate, utilize and transplant livers across the US.

## 1 Introduction

The landscape of liver transplant in the US has evolved considerably over the last 60 years since Dr. Starzl performed the first liver transplant (1). Advancements in perioperative management and surgical techniques have improved patient and graft survival. The demand for organs continues to outstrip the supply. The changing characteristics of a typical liver transplant recipient and a typical liver allograft donor in today's environment makes liver transplant a challenging endeavor. Despite the increasing number of transplants performed, the waitlist mortality continues to remain unacceptably high. Outcomes are challenged by older and sicker candidates and limited availability of high quality organs. As we continue to look into the future, it is important to understand this changing landscape and work on potential solutions to mitigate some of these challenges.

In this review we aim to specifically focus on the changing characteristics, in other words, increasing complexity of the liver transplant recipient, and the evolving characteristics of the typical donor. Our liver transplant recipients are becoming older, with multiple comorbidities, and increased burden of chronic and acute liver disease reflected in higher model for end stage liver disease (MELD) scores at transplant. The transplant event is magnified in complexity as deceased donors become less optimal in quality. We present a review of the literature on a road to a successful outcome in a high MELD recipient and how a “marginal” allograft could be successfully utilized in a high MELD recipient. We propose a more accurate description of the liver allograft quality using a concept of *Liver Allograft Variables* that utilizes some of the current liver donor risk index components and builds on it to reflect current landscape to produce a possible *Liver Allograft Composite Score*. We discuss how we could utilize this potential tool to generate more accurate and real time risk indexes and provide a framework of its application in our current era of liver transplantation. We conclude with our thoughts on potential future scenarios and their impact on liver transplantation in the US.

## 2 Changing characteristics of the liver transplant recipient

### 2.1 Changes in the etiology of end stage liver disease

**Figure 1:**
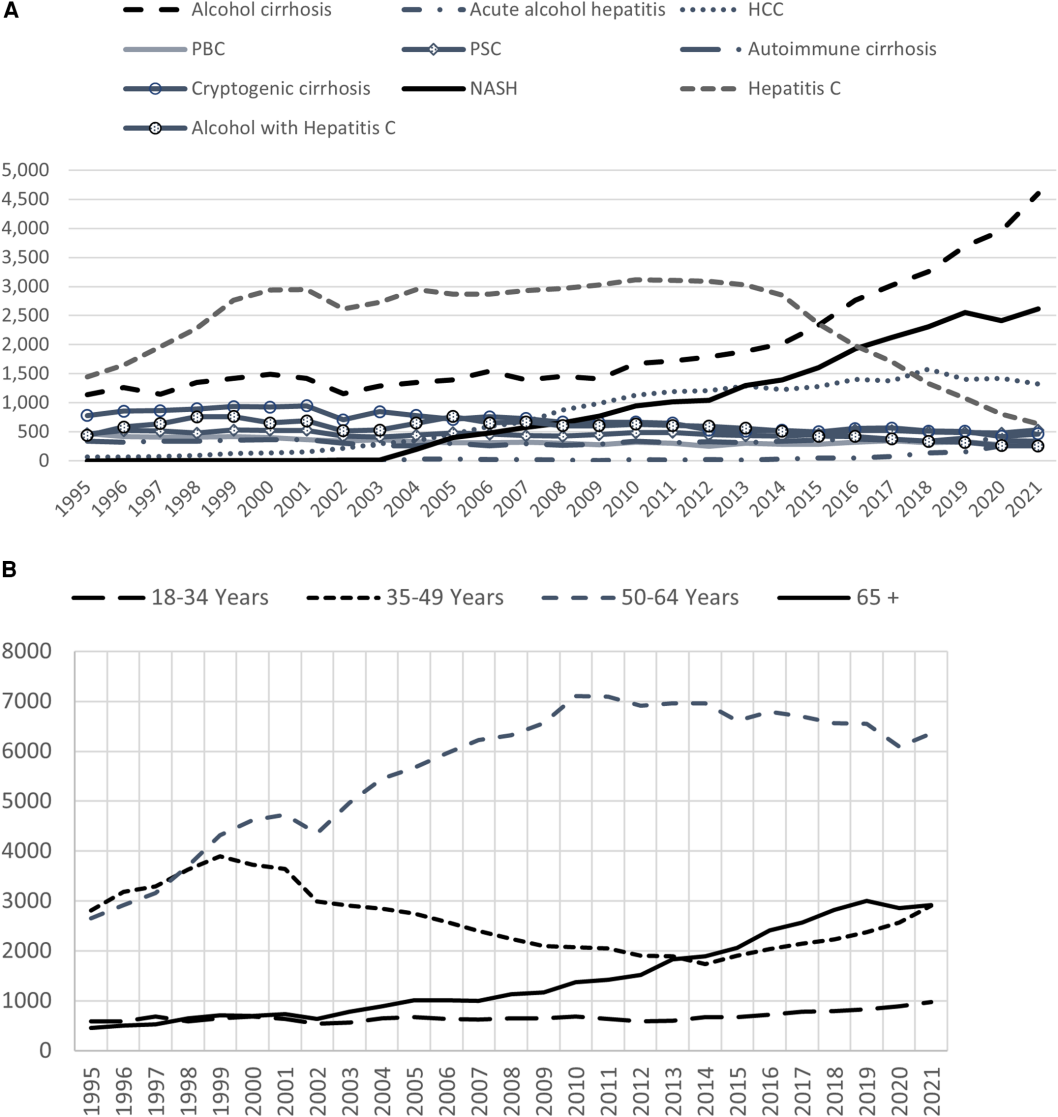
(**A**) Waitlist additions of new registrants by diagnosis. NASH and alcohol cirrhosis have become a leading diagnosis among the new candidates listed for liver transplant. Data available at the OPTN website was utilized (optn.transplant.hrsa.gov/data/view-data-reports/national-data) OPTN: Organ procurement and transplantation network. (**B**) Waitlist additions of new registrants by age. Age 50–64 cohort remains the leading group of new patients being added to the waitlist. Additionally, patients in 65+ age group have continued to rise over the past two decades and have surpassed the 35–49 age group over the past decade. Data available at the OPTN website was utilized (https://optn.transplant.hrsa.gov/data/view-data-reports/national-data). OPTN, organ procurement and transplantation network.

The main etiology of end stage liver disease leading to liver transplant has shown dramatic evolution over the past three decades. Review of the Scientific Registry of Transplant Recipients (SRTR) data from 1995 to 2021 shows significant increases in waitlist additions of new registrants with NASH and alcohol cirrhosis (Figure 1). Annual data report by the Organ Procurement and Transplantation Network (OPTN) shows that among adults, alcohol associated liver disease and NASH became the predominant indications for liver transplant (LT) in 2020 each comprising around 35% (2). Concurrently with this, the trend of new registrants with hepatitis C has sharply fallen over the past decade (Figure 1) as have the transplants done for this indication (2). This has been attributed to the availability of highly effective direct acting antivirals for hepatitis C within the last decade.

There has been an expansion in cancer indications for LT following the oncologic benefits seen in hepatocellular carcinoma (HCC). Currently, HCC and Cholangiocarcinoma are the only cancer indications that receive standardized MELD/PELD exception points. Other malignant indications including neuroendocrine and colorectal are gaining momentum (3–5). Criteria for utilizing living donors in these transplants are heavily institution based. Improvements will likely come from studies which can identify aggressive tumor biology that would not benefit from transplant (6), for instance, using circulating tumor DNA analysis. As we look into the future, it will be absolutely critical to study and scrutinize the role of downstaging, immunosuppression, biomarkers and innovative surgical approaches. Basic oncological principle of respecting tumor biology should govern ultimate decision making. Careful balance between maximizing equity and utility of available organs (deceased and living) and oncologic outcomes must be maintained.

### 2.2 Changes in the recipient age and comorbidities

**Figure 2:**
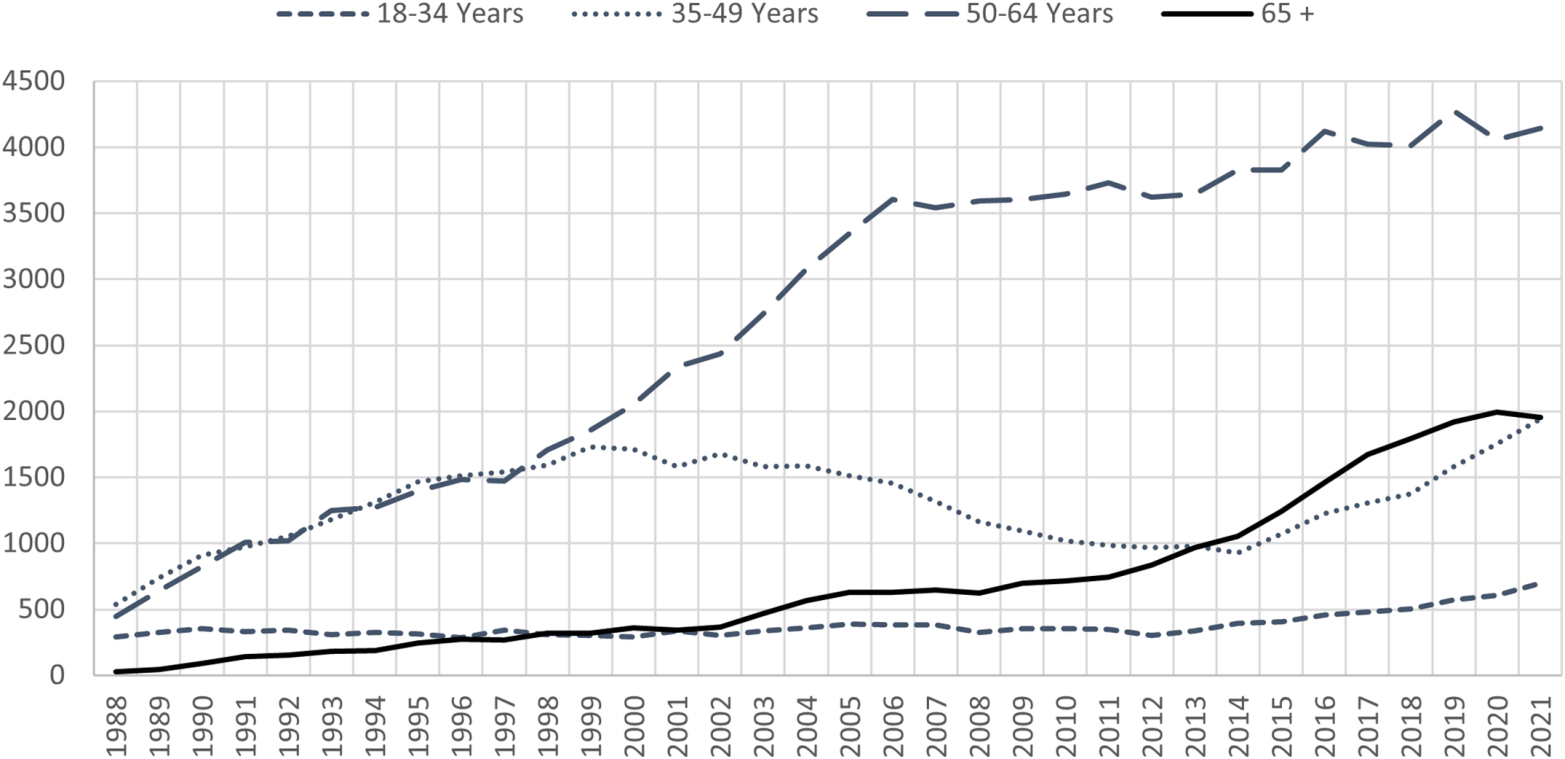
All liver transplants by recipient age. Vast majority of the transplants being done are in the 50–64 age group. Transplants in the age 65+ group are on the rise and have surpassed age 35–49 group in the past decade. Data available at the OPTN website was utilized (https://optn.transplant.hrsa.gov/data/view-data-reports/national-data). OPTN, organ procurement and transplantation network.

With the rise in obesity and diabetes, the incidence of non-alcoholic fatty liver disease (NAFLD) is on rise in the US. With this, non-alcoholic steatohepatitis (NASH) and liver related morbidity and mortality will increase in parallel. This is a unique population in which cardiac comorbidities and complications from diabetes are prevalent and challenge the transplant physician and surgeon. Patients with decompensated cirrhosis are at an even higher risk for sarcopenia, and malnutrition (7). Recent analysis of the OPTN data shows that the age 65+ group is the fastest growing cohort among the new registrants (Figure 1B) and transplants done in age group 50–64 and 65+ are on the rise (Figure 2). Coincident with the increased prevalence of NASH, 39.3% of recipients were obese (BMI > 30) and 28.9% had diabetes (2).

Additionally, frailty predicts waitlist mortality independent of MELD score with older candidates more likely to be frail with less physiological reserve (8). A recent frailty assessment called liver frailty index demonstrated that less than half of patients become “robust” after transplantation (9). Therefore, a strong emphasis needs to be placed on pre and post-transplant rehabilitation programs. Moreover, specific issues related to immunosuppressive therapy and kidney function warrant discussion. Immunosuppressive requirements to prevent rejection tend to lessen with increasing age, particularly for non-autoimmune conditions such as NASH and alcohol cirrhosis. Aging liver transplant recipients are at significant risk of chronic kidney disease due to preexisting kidney disease, metabolic factors and calcineurin inhibitor nephrotoxicity. Prevention and management of renal disease should follow established guidelines.

### 2.3 Changes in the disease severity

**Figure 3:**
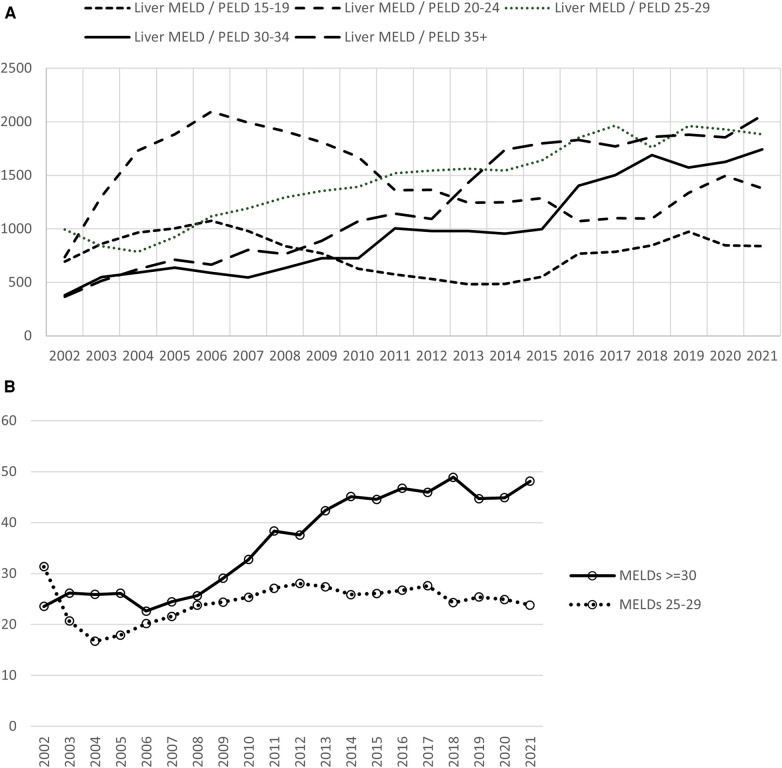
Deceased donor liver transplants according to MELD distribution. (**A**) Transplants for MELDs of 30 and above continue to rise. (**B**) In 2021, transplants for MELDs 30 and above comprised 48% of the transplants compared with 23% in 2002. A reduction in the transplants in MELDs 25–29 group from 2002 to 2021 was seen. Data available at the OPTN website was utilized (https://optn.transplant.hrsa.gov/data/view-data-reports/national-data). MELD, model for end stage liver disease; OPTN, organ procurement and transplantation network.

The increased demand for liver allografts combined with the stagnant supply has resulted in the continued increase in average MELD at transplant over the past two decades. OPTN/SRTR data from 2002 to 2021 shows the rising trend in the deceased donor transplants in MELD 30–34 and 35+  cohorts (Figure 3A). In 2002, MELDs 30+ group comprised 23% and MELDs 25–29 31% of deceased donor transplants done that year. In 2021, these numbers have dramatically changed with MELDs 30+ now making up 48% of all the deceased donor transplants. MELDs 25–29 subsequently decreased to 23.8% (Figure 3B).

As MELD scores continue to rise at transplant, more patients are at risk to develop acute on chronic liver failure. Acute on chronic liver failure (ACLF) occurs in 5%–30% of hospitalized patients with cirrhosis and has a mortality rate of 25%–42% at 28 days and 40%–56% at 90 days (10–12). Patients with ACLF-3 per European Association for the study of liver-chronic liver consortium criteria with three or more organ failure have poorer outcomes compared with less severe ACLF or patients with decompensated cirrhosis without ACLF. Liver transplant among select ACLF patients provides survival benefit (13, 14). A recent study (15) using United Network for Organ Sharing (UNOS) database on cohort of patients with ACLF-3 selected to receive LT aimed to develop a risk score for LT recipients and donor selection. Among recipients with a high-risk score, donor factors of age ≥60, grafts from national sharing and macrosteatosis >15% were associated with 1 year patient survival below 66% vs. 83% receiving higher quality allografts. Which grafts should and should not be used for these recipients continues to pose a challenging clinical dilemma.

## 3 Changing characteristics of the liver deceased donor

### 3.1 Age

Use of liver grafts from older donors has increased in recent years due to increased overall donor age and the prevailing organ shortage (16). Although there are changes that occur in the liver as a result of aging, the overall global functional decline is less than that in kidney or heart allografts (17, 18).

Recent study (19) assessing the trends in transplant of liver grafts from older donors (aged ≥ 70) and outcomes in recipients of these grafts suggests that these grafts may be underused. The adjusted OR for discard among older donors compared with younger donors over the study period was over 2. These grafts were used in recipients with mean age of 58 and mean laboratory MELD 18 at the time of transplant. Outcomes improved over time with 40% lower graft loss risk and a 41% lower mortality risk. These results were beyond the general temporal improvements in graft loss and mortality risk among recipients of liver grafts from younger donors. This shows these older grafts when properly selected and transplanted into appropriate recipients can be utilized successfully. Further opportunities to maximize their utilization are needed.

### 3.2 Steatosis in allografts

It is predicted that in US prevalence of obesity will increase to 48.9% and severe obesity (BMI ≥35) to 24.2% by the year 2030 (20). This obesity epidemic is expected to further increase the proportion of steatotic allografts. These grafts are particularly sensitive to preservation and ischemia reperfusion injury (IRI), increasing the risk of graft dysfunction (21). Compared to grafts with ≤5% steatosis, recipients undergoing LT with a graft having >30% macrosteatosis had longer intensive care and hospital stay and higher transfusion requirements (22, 23). Recent evidence has however demonstrated similar outcomes in long-term prognosis, biliary and vascular complications between moderate (30%–60%) and mild (<30%) (24, 25) macrosteatotic allografts.

### 3.3 Donation after cardiovascular death (DCD)

Livers procured for transplant from DCD donors experience more IRI and higher rates of ischemic cholangiopathy compared with donation after brain death (DBD) allografts due to period of warm ischemic time (WIT). Utilization of DCD livers has therefore traditionally been limited to short WITs and younger donors resulting in a higher discard rate compared to DBD donors. Inferior patient and graft survival following DCD LT contribute to persistent reluctance by centers to expand DCD acceptance criteria (26). More recently though, OPTN/SRTR data shows over the past decade there has been a consistent increase in the DCD livers transplanted (from 289 in 2009 to 831 in 2020, Figure 4). There is, however, still a high discard rate among DCD organs compared with DBD organs (<10% among DBD vs. close to 30% with DCD). Using national registry data, Scalea et al. (27) demonstrated that liver transplants from younger DCD donors (<50) had superior graft survival than transplants from older DBD donors (>60) suggesting that careful selection of DCD donors could increase DCD utilization without compromising outcomes. Ex vivo machine perfusion as well as normothermic regional perfusion (NRP) technology offers opportunities for assessment of viability and possibility of treatment of a “marginal” DCD without risk to recipient. As we embark on many machine perfusion related technologies that are starting to become mainstream in the clinical realm, this offers opportunities for utilization of this pool in ways we had not entertained before.

**Figure 4:**
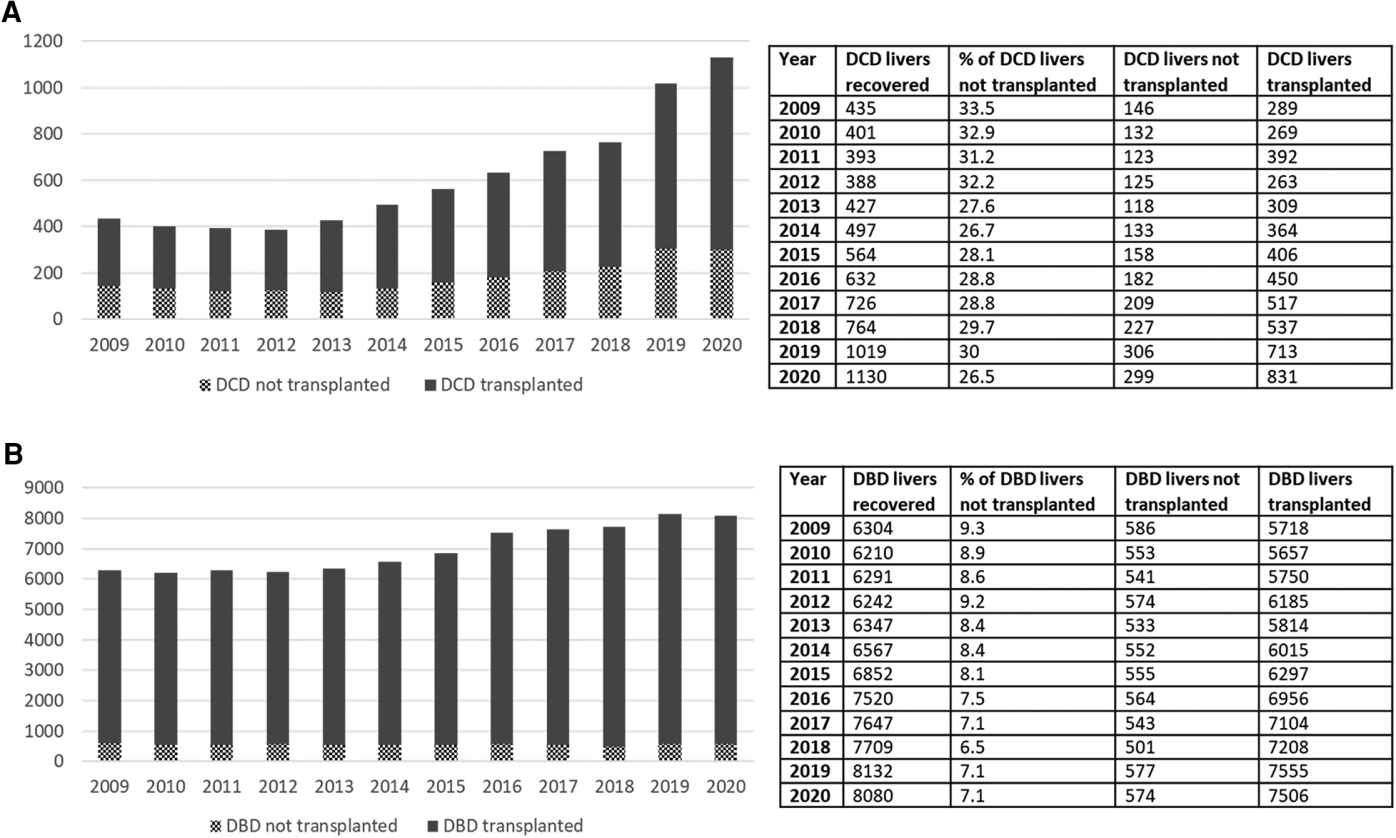
(**A**) Increase in the number of DCD transplants done over the past decade. (**B)** Relatively modest increase in the DBD transplants over the past decade. Rate of increase in DCD transplants over this time frame is much higher than the DBD transplants. Despite that, the rate of discard for DCD still remains unacceptably high. DCD, donation after cardiac death.

### 3.4 Hepatitis C positive grafts

The introduction of direct acting antiviral (DAA) therapy, with sustained virological response rates nearing 100% has changed the utilization patterns of livers from hepatitis C positive donors (28). Use of baseline donor liver biopsies have encouraged increased utilization safely. In the post DAA era, early graft and patient outcomes appear to be similar between recipients receiving HCV-positive and HCV-negative grafts (29).

## 4 Road to a successful transplant outcome in a high MELD recipient

There is a huge variation in the median MELD score in regions across the US. Present literature addressing liver transplant in high MELD recipients is scant with evaluation of these issues limited to single center studies. The heterogeneity of the recipient population and the practice patterns at various institutions make it extremely challenging to generalize results. This is reflected in the patient outcomes in various studies with one-year patient survival ranging from 72 to 89% with MELD scores ≥40 (30–32). Existing liver transplant infrastructure, donor selection or graft selection may need to be assessed and revamped in centers achieving lower survival rates.

### 4.1 Appropriate recipient selection

In this climate of organ shortage and sicker patients, appropriate patient selection will be important. A 50% 1-year post transplant survival has been documented for NASH recipients with age ≥60 years, BMI ≥30, diabetes and hypertension (29). Should this cohort be considered a contraindication for liver transplant? Given the comorbid conditions that often come with NASH induced cirrhosis, a multidisciplinary approach is required. This becomes even more important as we combine baseline comorbidities with higher degrees of native liver dysfunction, i.e., higher MELD patients.

### 4.2 Appropriate preoperative optimization and waitlist management

Waitlist mortality does differ by geography and is not clearly reflective of organ availability suggesting center behavior, referral and waitlist practices and patient management are varied around the country (33). Increased waitlist mortality has been reported in patients with high MELDs with severe hepatic encephalopathy (HE). In this large study (34), on a multivariate analysis, MELD ≥30 had a 58% greater risk of 90-day waitlist mortality than those without severe HE. There needs to be a timely transplant in this group of patients.

Centers differ greatly in their listing criteria and strategies. For example, some centers will wait until acutely ill high MELD patients stabilize before listing them for a transplant, while other centers may list sooner resulting in differential wait list death rates. Centers also differ when they choose to list patients with lower MELD scores. These low MELD patients do occasionally die on the wait list prior to receiving reasonable organ offers, therefore, centers who only list high MELD patients when stable for transplant may have lower relative wait list death rates.

### 4.3 Discussion and exclusion of futility

MELD prioritizes allocation to the “sickest” patients on the wait list. Patients with MELD ≥ 40 have the highest 3-month waitlist mortality rate (80%–100%) (35) but also receive the greatest survival benefit from LT (36, 37). Medical and economic efforts to bring these sicker patients with multiple comorbidities to and through LT are major challenges for every transplant center (38, 39). Petroswsky et al. (32) studied predictors of futility in recipients with lab MELDs ≥ 40. Futility was defined as 3-month or in-hospital mortality. Overall 1, 3, 5 and 8 year patient survival were 72%, 64%, 60% and 56%. They reported MELD score, pretransplant septic shock, cardiac risk, and comorbidities as independent predictors of futile outcome, rather than demographic, donor and operative factors. So called “extended criteria donors” defined as donor age ≥60, liver biopsy showing large droplet macrovesicular steatosis ≥20%, cold ischemia time (CIT) ≥ 10 h and/or WIT ≥ 60 min were utilized equally in both groups in the study with no significant difference. This, however, is a single center study with small number of patients in the MELD ≥40 cohort which does limit the generalizability of these results.

Other studies on the subject, Panchal et al. (31) and Evans et al. (40) have also shown inverse correlation between patient overall survival and increasing MELD score. Futility predictors included age >60, obesity, peritransplant intensive care unit (ICU) with ventilation and multiple comorbidities (31). As we continue to embark on transplanting this very sick cohort of patients, we need to consider the question of futility and continue studies to guide our informed decision making.

More specifically, benefits must be weighed against both greater risks and increased resource utilization. Patients with high MELD scores have been found to have increased incidences of post-transplant infection, longer ICU and general hospital stays and imply overall increased costs (38, 39).

### 4.4 Importance of Anesthesia personnel with experience in complex liver transplant cases

Patients with end stage liver disease (ESLD) have complex problems such as cirrhotic cardiomyopathy, coronary artery disease, hepatopulmonary syndrome, portopulmonary hypertension, hepatic encephalopathy, hyponatremia, hepatorenal syndrome and coagulopathies. Anesthesia management for these patients is dynamic, challenging, often requiring advanced monitoring such as transesophageal echocardiography and thromboelastography. Surgical techniques could include complete or partial occlusion of IVC with or without venovenous bypass or portocaval shunts. Post reperfusion syndrome is a crucial event where patients may experience arrhythmias or even cardiac arrest.

There is considerable evidence suggesting that dedicated liver transplant anesthesia care makes a significant difference in patient outcomes (41–43). Improved outcomes and fewer resource utilization was seen when anesthesia was delivered by a tightly knit team of physicians experienced in care of liver transplant patients (41). A recent study (44) by the Liver transplant anesthesia consortium found that academic liver transplant programs in the study had distinct anesthesia teams for liver transplants though their criteria for membership and responsibilities was inconsistent. Most conformity was seen on initiation of care. Fewer team members were involved in extended care of patients and were available for patients needing subsequent surgery. Trends in the data were associated with center volume with high volume centers utilizing on the job training for team membership which was rare at low volume centers. In comparison with larger teams, few low volume centers provided postoperative care or participated in activities that intersected with the larger multidisciplinary team.

### 4.5 Expert liver critical care management

In patients with higher MELD scores, perioperative care is inevitably at the level of intensive care. Studies have shown that patients with MELD 30 utilized ten times more Medicare spending than those with MELD 20 (45). Utilization of pre transplant dialysis was seen in nearly half of transplant patients with MELD ≥ 40 (31). This was mirrored in a Canadian study (46). Although 1- and 3-year patient survival was not significantly different in this study, graft survival was significantly inferior in the higher MELD group. Causes of graft failure were mainly arterial and biliary complications as “sicker” patients physiologically do not tolerate such complications well. In a study (47) published from a higher volume center transplanting primarily high MELDs, marginal/extended criteria donor (ECD) grafts were utilized for high acuity recipients (MELD ≥ 35) with similar short and long term patient survival compared to standard livers. No significant differences in graft survival between the two groups was seen.

There are well appreciated fundamental concepts in successful transplant of high MELDs. Larger volume centers that preferentially transplant sicker patients usually have more resources available at their disposal. Many such centers have liver patient dedicated ICUs which are managed primarily by liver transplant surgeons or liver focused intensivists in conjunction with pulmonologists. The availability of transplant fellows in larger programs to provide direct care in the ICU cannot be over emphasized. Nursing teams taking care of these patients routinely are well versed and experienced in managing issues unique to this patient population. Other organ specific specialties offer their expertise as needed with more routine knowledge of the ESLD patient. Additionally, in cases when a liver allograft is offered post cross clamp, larger centers have the bandwidth to accept these organs last minute and utilize them in their pool of recipients—whether they have more patients on a single matchrun, patients with different weights and sizes, sicker recipients that might already be in-house, available OR and anesthesia personnel with the ability of running simultaneous operating rooms and favorable logistics including possibility of placing some of these organs on machine pumps. With the deceased donor organ offer scheme now recently changed to distance circles as opposed to donor service areas, centers will have to face challenges not as often encountered previously.

The transplant center's volume experience cannot be understated. In 2020, 119 programs performed adult liver transplants with bottom quartile centers performing <25 per year, whereas the top quartile performing >100 liver transplants per year. A recent study (48) examined the clinical outcomes and resource utilization at low volume (<20/year), medium volume (20–25/year) and high volume (>55/year) centers. At low volume centers, less patients were listed, patients were less likely to receive a transplant, more likely to be removed from waitlist and associated with higher post LT mortality. This has borne out with living donor LT also (49).

With rising trends in higher MELD transplants and more non-standard organ offers, centers that can accomplish these transplants with successful outcomes will prevail whereas some of the low volume centers that are not as resource equipped will have challenges. In essence, it speaks to the notion that “rich” centers will continue to get “richer” and “smaller” centers will face more difficult challenges.

## 5 Successful use of a “*marginal*” *graft* in a high MELD recipient

Multiple terms are used to describe a “non-standard” liver allograft—“marginal”, “extended criteria donor”, “increased risk”, “high risk”. Although these are poorly defined and not standardized across studies, common characteristics include: donor age >60, hepatitis C positive donor, split livers, livers with extended cold ischemia time >12 h, DCD and macrosteatosis >30%.

Existing data in the literature on the use of non-standard liver grafts in high MELD population is very scarce. In this study by Amin et al. (50), survival was better with an immediate ECD transplant unless probability of primary non function (PNF) exceeded 23%, 72% and 88% for recipients with MELD scores of 11–20, 21–25 and 26–30 respectively. For patients with MELD scores >30, survival benefit with immediate ECD strategy persisted at even higher rates of PNF. Despite higher risk of PNF, transplant with an available ECD graft should be preferred over waiting for standard criteria donor (SCD) for patients with advanced MELD scores. Similarly, another study (47) showed that high acuity ECD graft recipients had similar short and long term patient survival (1-, 3-, 5-years) compared with standard liver recipients with no significant differences in graft survival or rejection free survival between the two groups. Recipients included in this study represented extremely ill cohort, the vast majority of whom were ICU dependent, requiring dialysis, mechanical ventilation, or life support treatment. Although these are single center studies, institutions that can not only provide successful transplant outcomes for higher acuity patients but at the same time utilize ECD organs in these patients become extremely important especially now in the current climate where more sicker patients are being listed and the average donor pool is becoming ever more non-standard. Therefore, consideration of an ECD graft use in high acuity patients should be weighed against the established morbidity and mortality risk of remaining on the waitlist.

In the era of machine perfusion technology gaining momentum, our terms and definitions of marginal organs need a revision. A number of questions should be considered, such as: (1) How should cold and warm ischemia time be defined? (2) What should be the assessment criteria for grafts placed on machines and the acceptable relevant viability and functional criteria while on the machine? This calls for reassessment of our current terminology and definitions. With the advent of direct acting antivirals with cure rates approaching 100%, is it now time to consider hepatitis C positive organs with acceptable procurement biopsies as standard grafts? Another important point that merits discussion is how we quantify the different possible components that define current “non-standard” organ terminology; how many of these individual components need to be present and in what severity to satisfy a definition of non-standard liver allograft? More likely though, each of these factors individually do not alone increase the risk of graft failure substantially, but rather the various combinations, especially at extremes probably portend a risk of bad outcome. Hence, it's time for us to now think about other tools that would be more clinically relevant in assessing the risks of potential non-pristine grafts. We suggest time is now ripe to look into devising nomograms that would consider gradation of these risk factors and subsequently yield results that we could more uniformly interpret and correlate to outcomes.

## 6 Time for a new innovative way to define and utilize the “non-standard liver allograft” (NSLA)—a proposal for a new concept termed *liver allograft composite score (LACS)*

**Figure 5:**
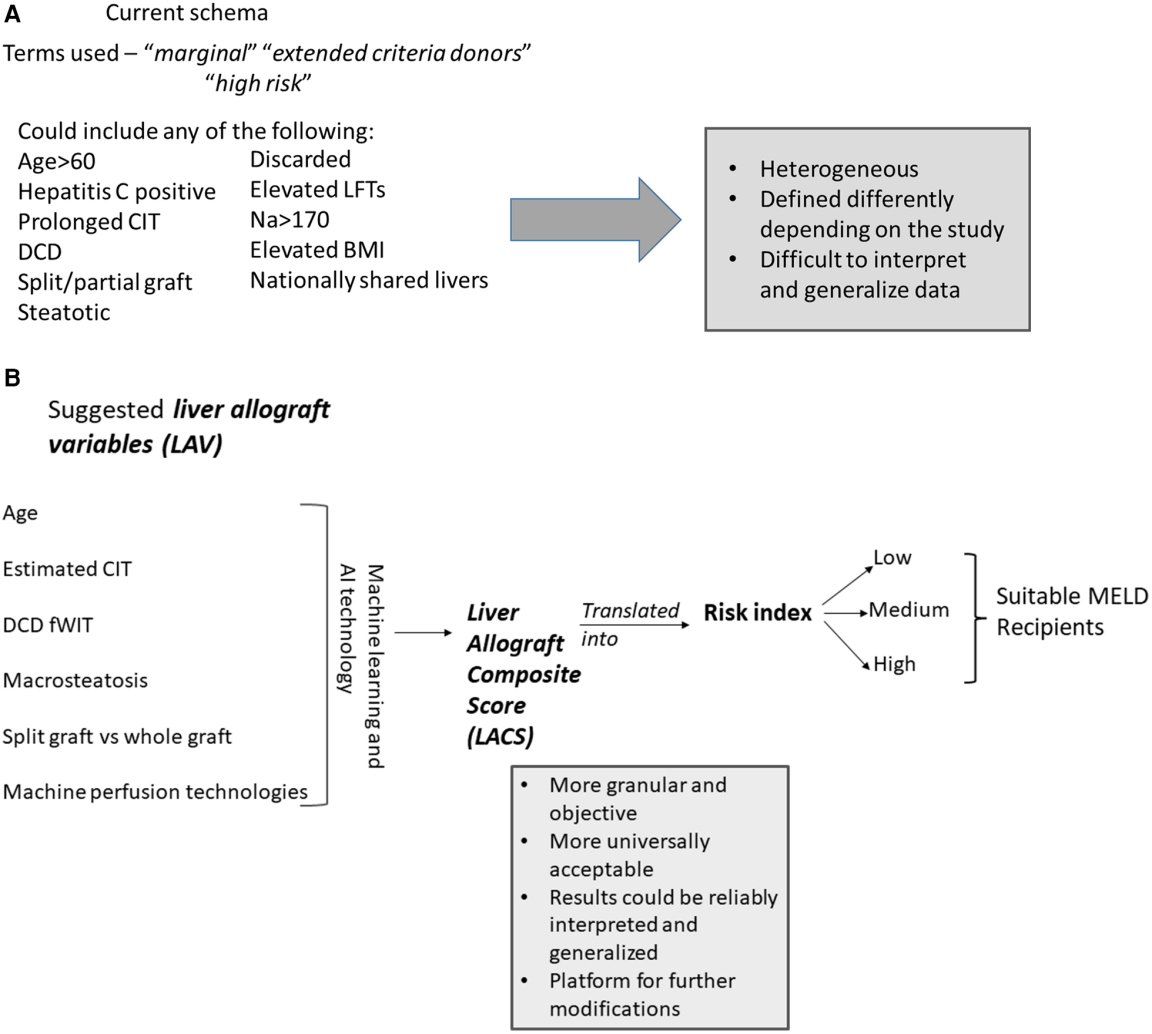
(**A**) Current description of non-standard donor liver allograft. Multiple terms are utilized and there is great heterogeneity in the concept of non-standard liver allograft. (**B**) New proposed schema—introduction to the concept of “*liver allograft variables*” and “*liver allograft composite score*”. Various suggested variables form the backbone of this strategy, which with machine learning and AI technology will formulate a “*liver allograft composite score*” which could then be translated into various risk indexes suitable for relevant MELD recipients. (**C**) Suggested schema outlining liver allograft utilization based on *liver allograft composite score* and risk index. Based on the various risk indexes, an allograft would be either utilized as such for the appropriate MELD recipient or would be intervened upon reducing the risk index and then utilized in the appropriate MELD recipient. (**D**) Alpha numeric representation of the *liver allograft variables* defining an allograft at a time point. The unique alpha numeric code would identify a particular allograft pre and post intervention which would inform real time decision making. AI, artificial intelligence; CIT, cold ischemia time; DCD, donation after cardiac death; fWIT, functional warm ischemia time; L, left; LFTs, liver function tests; LLS, left lateral section; MELD, model for end stage liver disease; R, right; RI, Risk index; WIT, warm ischemia time.

In the quest to address the unacceptably high waitlist mortality, expansion of the donor pool presents as an immediate viable solution. This has led to the evaluation of “marginal”/“ECD” allografts, poorly defined terms over decades that have generally included grafts from older donors, hepatitis C positive donors, donation after cardiovascular death, grafts with prolonged cold ischemia time, steatotic grafts and split grafts (51). These allografts were collectively termed as such because of past studies showing an increased risk of graft failure and poor outcomes (51–53). There is no consensus definition for marginal/ECD livers. In addition to the criteria above, some studies include discarded (initially declined for transplant), nationally shared livers, donors with serum Na > 170, elevated liver enzymes and/or high BMI to be marginal livers (51, 54) (Figure 5A). This further adds to the ever pervasive heterogenous definition of marginal/ECD livers in the literature making it extremely difficult to interpret, understand and generalize outcomes. Additionally, it is very difficult to define a standard liver graft. With rising obesity and increased alcohol use, some of the allografts from younger donors are also severely steatotic. With widespread popularity and utilization of machine perfusion techniques in organ procurement and preservation across the country, the nomenclature of standard vs. non-standard graft is becoming more blurry. Prior liver donor indexes such as the donor risk index for liver transplant (LDRI) developed almost two decades ago by Feng et al. are not considered practical by many nor are widely utilized in the clinical realm (52). Hence, there is a grave need to revisit this concept in the current clinical landscape and formulate models that are comprehensive, practical, reliable, easy to use universally and can change in real time depending on changing characteristics of the allograft.

**Figure:**
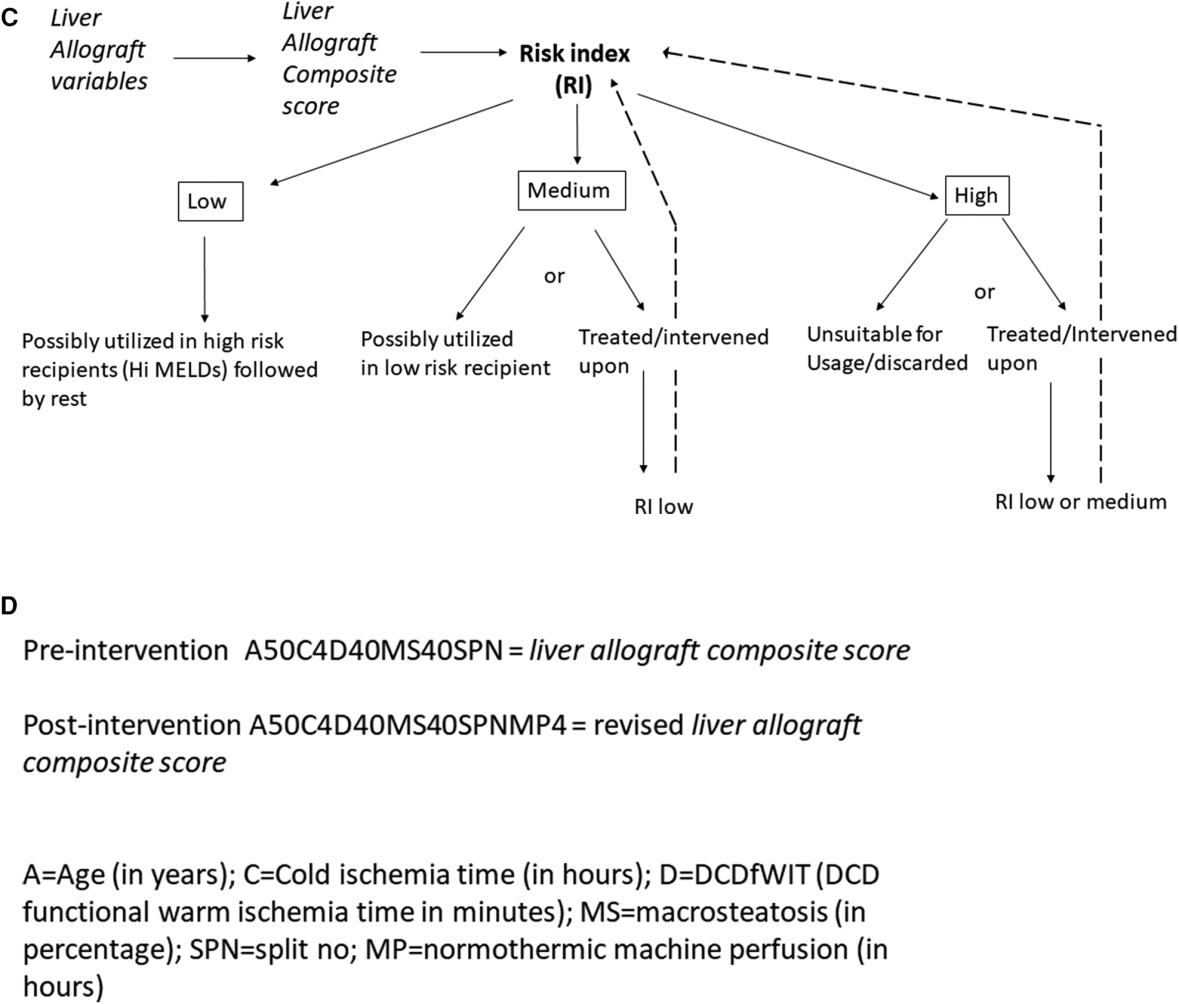


We introduce and propose a new schema (Figure 5B) which incorporates components of the previously validated LDRI and adds additional relevant factors known at the time of the liver offer as well as factors which could be manipulated in the future such as machine preservation, that we term as *liver allograft variables* which could combine to form a numeric *liver allograft composite score*. We suggest the following factors be considered during its initial version—donor age, anticipated/actual CIT, DCD with spectrum of functional warm ischemia time [with provisions for modification due to normothermic regional perfusion (NRP)], level of macrosteatosis, whether the graft is whole or split, and various machine perfusion techniques (normothermic, hypothermic, and combinations). We propose a machine learning or artificial intelligence (AI) defined calculation in which these and other potential variables could be combined into a score which would be available to the clinician accepting the liver offer. The clinician could then change future factors—such as adding NRP prior to crossclamp or normothermic machine perfusion (NMP) after procurement to decide which modalities may be useful to optimize the function of this specific allograft for her/his recipient's needs. We further propose that a consensus conference of experts be put together to discuss these components in a more detailed and granular way. This includes discussion regarding which variables to initially include and exclude, whether a large amount of raw data is evaluated by machine learning/AI to create the score initially, or if a set of estimated initial weights should be calculated/assigned to each variable. In the future, there should be consideration to align the composite score to the appropriate MELD groups for optimal use of all donated allografts. The mathematical analysis and computation of these scores would be further enhanced by the ongoing use of artificial intelligence and potential relevant softwares that could be developed for this purpose. This process of creating a score to encompass many more granular details of the allograft before, during, and after procurement would be very objective and would remove the ambiguity that currently exists in the literature.

The score could be eventually translated into a risk index which would define a graft at a particular moment in time (Figure 5C). Risk indexes could describe the risk of mortality, primary non function, early allograft dysfunction, ischemic cholangiopathy to name a few. If a risk index is deemed too high for a potential recipient by that accepting clinician, the clinician could consider some form(s) of intervention for this graft such as machine perfusion technology to bring the risk index to a more acceptable level. If this graft could not be improved to an acceptable level, that clinician may pass on that organ for that recipient. In other words, this proposal would allow us to start objectively thinking about what organs could truly benefit from intervention(s), which intervention(s) and which grafts do not require interventions prior to implantation. Over time with enough data, a machine learning algorithm could suggest to the clinician, the appropriate technology(ies) to bring the risk index low enough to be safely utilized in patients in various MELD categories. This could revolutionize the way we approach organ utilization. It could lead to a system that is very objective, maximizes organ utilization safely and will be instrumental in mitigating waitlist mortality and organ discard. As new data and literature becomes available and more robust, the platform could be modified appropriately to reflect the new available evidence and improve practice. Likely not all grafts need to be intervened upon prior to usage. The individual scores and risk index would allow us to track the allograft at different stages for eventual utilization in the appropriate recipient. All of the information would be available in real time and could be applied in real time. In addition to the *composite score*, we suggest that an alpha numeric sequence of the data be recorded (Figure 5D). This would greatly help with having the real data readily available that can then be utilized to inform decision making. This would be important for future research projects that would allow us to access the relevant key data as it changes over time for a specific organ. It, therefore, becomes extremely important that OPTN develops systems to capture this data moving forward.

To provide an example—consider the following offer of a 52-year-old male, DCD with 40 min fWIT (defined as systolic BP < 80 or O2 saturation <80%—a very conservative definition which could change overtime), 30% macrosteatosis, estimated CIT 5 h. Lets assume that this yields a *composite score* and a corresponding risk index that is unacceptably high. However, if with NRP the risk index drops down enough, it could potentially be safely utilized in a mid-level MELD score recipient if appropriate. Rather than offering this organ as is, it might be prudent to plan to do NRP in this case. Similarly, consider the following case of a 26-year-old female, DCD with 10 min fWIT, no macrosteatosis and estimated CIT 5 h. If this yields a *composite score* and a corresponding risk index that is low enough to be utilized in a mid-level MELD score, the graft likely does not require any interventions prior to usage and can be offered as such. Groups of MELD scores could be imagined that would be appropriate for a certain risk index level and if too high a risk, then either the organ is treated to decrease the risk and then could be offered to that MELD group, or it could be offered without treatment to a lower MELD group with an acceptable calculated risk without allograft intervention. Checks and balances would need to be added to the system to make sure principles of equity, need and fairness are respected.

We fully recognize that the schema that we are highlighting here is purely hypothetical. However, the foundational concepts and thoughts are based on emerging clinical data being published. Recent multicenter study in the US involving NRP technology has shown reduced rates of ischemic cholangiopathy, biliary complications and early liver allograft function in NRP recipients compared with standard super rapid recovery (55). This has also borne true in other allografts. A large national US study recently reported improved early post-transplant outcomes and organ use in kidney transplants using NRP for DCD (56).

The conversion of this schema into a working practical clinical model will require much work. But we feel that the time is ripe now to start thinking in those directions. Multiple work groups, teams and stakeholders would need to be considered and first steps should include discussion of the current state of organ allocation, emerging technologies and tremendous amount of data being generated and published. The basic question to ask here would be how do we take all this information and convert it into a practical, clinically applicable platform that would provide a framework for incorporating donor characteristics, application of technologies to organs in real time, recipient characteristics and help with decision making for organ allocation in real time. Our schema is one way of processing those thoughts and provides a starting place to begin discussions on the topic. Creation of an expert panel conference to work on different elements of this would be essential. Much work, particularly accurate donor, procurement and allograft treatment data collection, as well as more detailed patient/graft outcome data collection, would need to be done upfront when establishing the basis for the calculations and creating a foundational platform However, once the platform is created it would serve as a foundation for many years to come and will continuously learn with each new transplant and addition of any new technology. We fully recognize that the variables incorporated in the calculations will likely change over time, but the platform would provide a much needed infrastructure upon which to construct further iterations of this schema. An expert panel group would be involved in constant revisions and modifications with the help of artificial intelligence technology to keep the system running and up-to-date. We feel that this could be the optimal future of organ allocation. It allows the OPTN to constantly be at the verge of the new data application to practice, in real time and continue to serve our patients in dire need of these life-saving organs. Simultaneously, the data collected in real time would continue to refine the practice patterns and would serve as a positive feedback loop. This truly has the potential to innovate and improve the way we allocate, utilize and transplant livers across the nation. The concept is also expandable to be used in any organ allocation system in the world, and for any organ incorporating that specific organ's important characteristics and any relevant treatment technologies.

## 7 Conclusion

The landscape of liver transplantation in the US is in evolution. We have seen dramatic changes in the characteristics of the donor as well as the recipient pool. The recipient pool is becoming sicker, with multiple significant comorbidities and with higher MELDs being listed and transplanted. At the same time, the donor pool continues to become more “non-standard/marginal”. Couple this with the explosion of machine perfusion technologies swiftly gaining momentum across the country, our fundamental understanding of the so called “non-standard liver allograft” requires our much needed revised attention. Our proposal of a more objective way of addressing this issue by introducing an idea of a score that we term as “*liver allograft composite score*” and proposed associated risk index is one such step in that direction. It recognizes that multiple variables including inclusion of machine perfusion techniques need to be considered at organ offer level. It is more than likely that not all organs need to be treated prior to offering yet some grafts cannot be offered without appropriate treatment to reduce the so called “risk index” to an acceptable level for the recipient. This brings attention to the concept that we need to define a risk index level for appropriate MELD scores and at the same time consider the low to mid-level MELD score patients that are currently disadvantaged in our MELD system that is based on the acuity defined by current parameters. Application of these revised concepts could be instrumental in optimization of organ allocation, judicious utilization of new emerging organ perfusion/preservation technologies with emphasis on enhanced utilization of organs to all listed patients at every MELD score level and ultimately grow and further mature transplantation across the US.
